# A Real-Time Monitoring System to Assess the Platelet Aggregatory Capacity of Components of a Tissue-Engineered Blood Vessel Wall

**DOI:** 10.1089/ten.tec.2015.0582

**Published:** 2016-06-24

**Authors:** Faiza Idris Musa, Alan G.S. Harper, Ying Yang

**Affiliations:** Institute for Science and Technology in Medicine, School of Medicine, Keele University, Stoke-on-Trent, United Kingdom.

## Abstract

Native blood vessels contain both an antiaggregatory intimal layer, which prevents platelet activation in the intact vessel, and a proaggregatory medial layer, which stimulates platelet aggregation upon vascular damage. Yet, current techniques for assessing the functional properties of tissue-engineered blood vessels may not be able to assess the relative effectiveness of both these pro- and antiaggregatory properties of the vessel construct. In this study, we present a novel technique for quantitatively assessing the pro- and antiaggregatory properties of different three-dimensional blood vessel constructs made using a layered fabrication method. This technique utilizes real-time measurements of cytosolic Ca^2+^ signaling to assess platelet activation in fluorescently labeled human platelet suspensions using fluorescence spectrofluorimetry, while also permitting examination of thrombus formation upon the surface of the construct using fluorescent imaging of DiOC_6_-labeled platelets. Experiments using this method demonstrated that type I collagen hydrogels, commonly used as scaffolds for vascular tissue engineering, were unable to support significant platelet activation, while type I and III neo-collagen secreted from human coronary artery smooth muscle cells cultured within these hydrogels as the medial layer were able to support thrombus formation. The incorporation of an intimal layer consisting of human umbilical vein endothelial cells on top of the medial layer inhibited platelet activation and aggregation. These data demonstrate that the methodology presented here is able to quantitatively compare the capacity of different constructs to trigger or prevent platelet activation. As such, this technique may provide a useful tool for standardizing the assessment of the functional properties of tissue-engineered blood vessel constructs developed using different culturing techniques.

## Introduction

The integrity of the high-pressure closed circulatory system of mammals is constantly maintained by a hemostatic system aiming to prevent excessive blood loss upon vascular injury.^1^ However, unwanted or uncontrolled activation of the hemostatic system can lead to a number of life-threatening cardiovascular diseases. Through studying the processes, which regulate platelet activation in (patho)physiological conditions, it should be possible to better prevent and treat patients suffering from these cardiovascular disorders.^2^ Currently, the favored research system in which to study *in vivo* thrombus formation is through the use of intravital microscopy to study clotting elicited by damage to the mouse mesenteric artery.^3–5^ However, there are many known differences in both the platelet transciptome and proteome, as well as the hemodynamics in mice and humans.^6^ In addition, intravital microscopy requires the use of general anesthetics, which have been shown to interfere with various aspects of blood clotting in previous *in vitro* studies.^7^ Thus, there appears a strong need to create a physiologically relevant *ex vivo* human blood vessel model to study the normal processes of thrombus formation. In this study, we hypothesized that a three-dimensional (3D) tissue-engineered human blood vessel construct may provide a useful model system to complement current animal studies of thrombus formation.

To create a human blood vessel construct for the study of hemostasis, it is imperative that it recreates both the key structural and functional features of native vessels. With regard to hemostasis, the key layers of the blood vessel are the *tunica intima* and *tunica media.*^8^ The *tunica intima* is an endothelial monolayer, which prevents platelet activation by acting as a physical barrier between the plasma and the underlying subendothelial matrix, as well as through the endothelial production of platelet-inhibiting paracrine agents.^9^ However, upon damage to the vessel wall, platelets become activated by a proaggregatory medial layer, through their adhesion to ligands present within the subendothelial matrix, of which collagen is the main ligand. The adhesion of platelets to the subendothelial matrix triggers their activation leading to thrombus formation, which prevents further blood loss.^10^

Advanced tissue engineering techniques have created the opportunity to produce a blood vessel construct with structural similarity to its native counterpart. However, currently, there is no sensitive technique to assess the relative effect of both the pro- and antiaggregatory functions of the vessel constructs. A common approach of assessing platelet activation on the surface of tissue-engineered vascular constructs is to examine platelet aggregation.^11^ However, sustained aggregation of platelets requires strong activation of platelets^12^ and so may not be sensitive enough to detect low-level platelet activation. Furthermore, the antiaggregatory properties of the tissue-engineered blood vessel constructs are usually assessed by examining nitric oxide (NO) or prostacyclin (PGI2) production by the endothelial cell, without examining their effect on platelets exposed to them.^9^ The lack of a technique able to assess the relative strength of these competing pro- and antiaggregatory properties of the blood vessels means that investigators cannot be certain whether any platelet aggregation observed is due to a failure of endothelial cells to inhibit platelet activity or due to the exposure of platelets to proaggregatory stimuli. Similarly, a lack of platelet aggregation may be due to overactive platelet inhibition by endothelial cells or failure of proaggregatory ligands to trigger effective platelet activation. Thus, there appears to be a need for a simple technique able to quantitatively assess the relative pro- and antiaggregatory effects of blood vessel constructs when exposed to platelets.

It is well known that cytosolic Ca^2+^ concentration ([Ca^2+^]_cyt_) measurements provide a sensitive method to effectively assess the activation state of platelets.^13,14^ All stages of platelet aggregation require a rise in cytosolic Ca^2+^ concentration—from the small amplitude, transient Ca^2+^ rises, seen in platelets that only transiently adhere to the damaged vessel wall; the moderate amplitude, sustained increases, seen in platelets irreversibly adhering and aggregating upon the vessel; to the high amplitude, prolonged increases, observed in phosphatidylserine-exposing procoagulant platelets.^15^ In addition, agonist-evoked cytosolic Ca^2+^ rises are strongly inhibited by the endothelial-derived antiaggregatory compounds, NO and PGI_2_.^16,17^ Thus, through monitoring [Ca^2+^]_cyt_ in real-time in human platelet suspension exposed to tissue-engineered vessel constructs, we may be able to sensitively and quantitatively assess the relative effects of the pro- and antiaggregatory effects of the constructs—hence offering a sensitive testing platform, which could provide a standardized system to directly compare the anti- and proaggregatory capacity of different tissue-engineered blood vessels. In this article, we report how we built and used this novel methodology to assess the relative ability of our full and partial tissue-engineered blood vessel constructs to facilitate or inhibit platelet aggregation.

## Materials and Methods

### Materials

Poly-l,d-lactic acid (96% l/4% d) (PLA) was purchased from Purac BV (Gorinchem, the Netherlands). Rat tail collagen type I was obtained from BD Biosciences. Mouse anti-human collagen type I and III IgG primary antibodies and goat anti-mouse IgG FITC were obtained from Santa Cruz Biotechnology. Primary human coronary artery smooth muscle cells (HCASMCs), human umbilical vein endothelial cells (HUVECs) pooled, medium 200, medium 231, low-serum growth supplement (LSGS), smooth muscle growth supplement (SMGS), cell tracker (CMAC), and 5-(and-6)-carboxyfluorescein diacetate, succinimidyl ester, mixed isomers (CFSE) were all obtained from GIBCO, Life Technologies. Fura-2/AM and prostaglandin I_2_ were both purchased from Cambridge Biosciences. Thrombin was obtained from Merck Chemicals. Chloroform, dimethyl formamide (DMF), rhodamine B, Sudan black B, Tween 20, fibronectin from bovine plasma, 3,3′-dihexyloxacarbocyanine iodide (DiOC_6_), and apyrase were all purchased from Sigma Aldrich.

### Electrospinning of PLA nanofibers

Highly aligned PLA nanofiber meshes were fabricated following a previously established laboratory protocol.^18^ To aid in visualization of the nanofibers as well as observation of the intimal and medial layers of the construct using the confocal microscope, rhodamine B was added to the 2% PLA solution at a final concentration of 0.1% (w/v) for fabrication of fluorescently labeled nanofibers. Following electrospinning, the deposited aligned nanofibers were transferred to cellulose acetate frames into portable and low line density-aligned nanofiber meshes. They were sterilized under ultraviolet light for 90 s three times before usage.

### Cell culture of primary cells

HCASMCs and HUVECs were cultured according to the supplier's recommendations. HUVECs were cultured in medium 200 supplemented with LSGS, whereas HCASMCs were cultured in medium 231 supplemented with SMGS. The cells were placed in a humidified incubator at 37°C and 5% CO_2_ until 90% confluent. In the present study, both HCASMCs and HUVECs were used until passage 5.

### Fabrication of multilayered tissue engineering blood vessel constructs

#### Construction of a three dimensional (3D) tissue-engineered medial layer

All reagents were kept prechilled on ice to prevent unnecessary gelation during preparation. HCASMCs were mixed at a density of 5 × 10^5^ cells/mL into a neutralized solution of 3 mg/mL type I collagen obtained from BD biosciences, according to the manufacturer's protocol; 0.2 mL of the mixture was then loaded onto a 1 cm^2^ square-shaped filter paper frame, and the collagen–cell mixture was left to set for 40 min at 37°C, 5% CO_2_. The formed gel was then covered with supplemented medium and media changed every 2 days. HCASMCs were subsequently allowed to grow in the tissue-engineered medial layer (TEML) until they were observed to possess an elongated spindle-shaped morphology.

#### Construction of a 3D tissue-engineered intimal layer

0.2 mL neutralized 3 mg/mL BD type I collagen solution was loaded upon a square frame made by filter paper with an area of 1 cm^2^ to form an acellular collagen gel base first. An aligned, fibronectin-coated PLA nanofiber mesh was prepared, which was then placed atop the constructed collagen gel. The coating was performed by placing the portable nanofiber mesh atop a nonadhesive PTFE plate. A 1 mL fibronectin solution with a concentration of 10 ng/mL was applied on these nanofibers and allowed to incubate for 1 h at room temperature. Following incubation, the fibronectin solution was removed, and the mesh was dried and used for construct fabrication. After successful attachment of the nanofibers to the collagen hydrogel, HUVECs were then seeded on top of the nanofibers (4 × 10^4^ cells per sample). The cells were allowed to attach at 37°C, 5% CO_2_, for 1 h. The formed samples, tissue engineered intimal layer (TEIL), were then topped up with supplemented 200 medium and subsequently allowed to be cultured for 4 days at 37°C and 5% CO_2_, with a single media change on day 2.

#### Construction of a 3D multilayered tissue engineering blood vessel

A 3D multiple layered tissue engineering blood vessel (TEBV) construct was fabricated by using combinatorial procedures of the 3D TEML (Construction of a 3D tissue-engineered medial layer section) and intimal layer (Construction of a 3D tissue-engineered intimal layer section), that is, incorporating the intimal layer atop TEML construct. In detail, once the HCASMCs acquired a spindle-shaped morphology in TEML, a nanofiber mesh coated with fibronectin was placed to create the initial layer of the luminal surface. HUVECs were then seeded on top of the nanofibers as stated in the Construction of a 3D tissue-engineered intimal layer section. Samples were then topped up with the mixture supplemented medium 200 and 231 at the ratio of 7:3, and subsequently allowed to be cultured for 4 days at 37°C and 5% CO_2_, with a single media change on day 2. To confirm the multilayered structure of TEBV and the location of the associated cells, individual layer was stained with different fluorescence dyes. HCASMCs were labeled with CFSE (80 μM) for 15 min at 37°C and 5% CO_2_ before seeding into the hydrogel. HUVECs were labeled with CMAC cell tracker (20 μM) for 30 min at 37°C and 5% CO_2_ before seeding atop the nanofiber. The cells were washed and resuspended with a fresh medium to remove the excess dye after the end of incubation and before seeding.

### Platelet preparation

This study was approved by the Keele University Research Ethics Committee. Blood was donated by healthy medication-free volunteers who gave written informed consent. Blood was collected by venipuncture and mixed with one-sixth volume of acid citrate dextrose (ACD) anticoagulant (85 mM sodium citrate, 78 mM citric acid, and 111 mM d-glucose). Platelet-rich plasma (PRP) and washed platelets were prepared following well-established protocols.^14^ In detail, PRP was prepared by centrifugation for 8 min at 1500 *g* before aspirin (100 μM) and apyrase (0.1 U/mL) were added. Washed platelet suspensions were obtained by centrifugation of PRP at 350 *g* for 20 min to attain a cell pellet, which was then resuspended in HEPES-buffered saline (HBS; pH 7.4, 145 mM NaCl, 10 mM HEPES, 10 mM d-glucose, 5 mM KCl, and 1 mM MgSO_4_), as previously described.^19,20^ The HBS was presupplemented with 1 mg/mL bovine serum albumin, 10 mM glucose, 0.1 U/mL apyrase, and 200 μM CaCl_2_ (supplemented HBS). Extracellular Ca^2+^ concentration was raised to 1 mM immediately before the start of the experiment. The cell density of the washed platelet suspension was then adjusted to 2 × 10^8^ cells/mL by addition of further supplemented HBS in all experiments.

In some experiments, platelets were labeled with the fluorescent membrane dye, DiOC_6_, and the cytosolic calcium indicator, Fura-2/AM, to allow us to examine platelet activation both on the surface of the construct and in the platelet suspension. Whole blood was collected into ACD containing DiOC_6_ at a final concentration of 1 μM. The blood was mixed with anticoagulant and dye and left to incubate for 10 min at room temperature before centrifugation. The collected PRP was then treated with aspirin and apyrase as stated above and incubated with 2.5 μM Fura-2/AM for 45 min at 37°C. Platelets were then washed by centrifugation and resuspended in supplemented HBS as stated above.

### Examining pro- and antiaggregatory properties of the 3D vessel constructs

#### Measurement of platelets' cytosolic Ca^2+^ concentration ([Ca^2+^]_cyt_) when exposed to 3D models

**FIG. 1.**
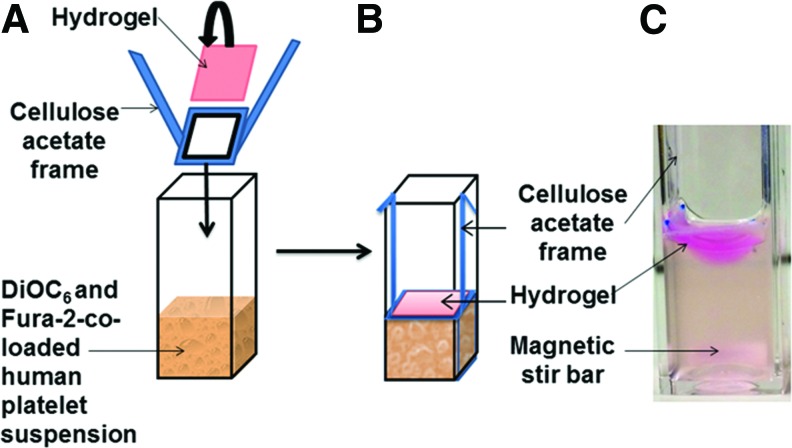
The pictures showing the novel testing platform to assess the proaggregatory capacity of acellular collagen hydrogel or tissue-engineered vessel constructs in the *ex vivo* platelet Ca^2+^ signaling assay. **(A)** The three-dimensional sample to be tested was placed on a cellulose acetate frame with the center part permitting direct exposure to Fura-2- and DiOC_6_-co-labeled washed human platelet suspension. **(B)** The sample was lowered down, allowing contact with the platelet suspension. **(C)** The picture of the assembled test sample with the presence of constant stirring of platelet suspension using a magnetic stir bar shown at the *bottom*. Platelet Ca^2+^ signaling in the platelet suspension was measured in real-time through Fura-2 fluorescence intensity, while platelet adhesion and aggregation on the surface of the samples were assessed by the imaging of DiOC_6_-labeled platelets after removal of the sample from the cuvette. Color images available online at www.liebertpub.com/tec

Fura-2 fluorescence measurement was recorded from 1.2 mL dually labeled washed platelets in cuvettes with constant magnetic stirring. The constructs (TEML, TEIL, TEBV, and acellular collagen gel) were placed on a sample holder comprising a cellulose acetate frame with the central window permitting exposure to washed platelet samples when it was lowered into position (Fig. 1A, B). After 15 min of incubation at 37°C, the construct was removed for imaging and the remaining platelet suspension was then stimulated with 0.2 U/mL thrombin and monitored for a further 5 min. Fura-2 fluorescence was continuously recorded using a spectrophotometer (Cairn Research) using excitation wavelengths of 340 and 380 nm and emission of 515 nm. Changes in [Ca^2+^]_cyt_ were calculated using the 340/380 nm fluorescence ratio and calibrated according to the method of Grynkiewicz *et al.*^19^ Agonist-evoked changes in [Ca^2+^]_cyt_ were quantified by integration of the change in fluorescence records from basal with respect to time for 15 min postconstruct addition and/or 5 min post-thrombin addition.

#### Imaging of DiOC_6_ fluorescence on the luminal surface of blood vessel constructs

Washed DiOC_6_-labeled platelets were incubated with the vessel constructs and acellular collagen gel, as a control, for 15 min at 37°C with continuous stirring. At the end of the incubation period, constructs were removed from the platelet samples, washed with HBS, and imaged under a fluorescence inverted microscope (Nikon) with an excitation wavelength of 485 nm and emission of 501 nm.

### Collagen morphology assessment

Acellular collagen hydrogels and cultured TEML were transferred onto Petri dishes with centrally located coverslips to enable visualization of their fibril morphology using a confocal microscope (FV1200; Olympus) under reflection mode.

For imaging using scanning electron microscopy (SEM), the same group samples were subjected to standard critical point drying first. SEM examination used a bench top Hitachi TM3000 system under 1.5–5 kV acceleration voltage.

### Immunohistochemical staining of human type I and III collagen fibrils

The presence of type I and III collagen of the TEML constructs was examined using monoclonal antibodies specific to human type I and III collagen, respectively. TEML constructs cultured for 10 days were fixed for 40 min at room temperature with 4% paraformaldehyde. Subsequent to fixing, the TEML constructs were incubated with Sudan black B for 30 min at room temperature. This was followed by blocking with 5% goat serum for 30 min at room temperature. The cells were then incubated with either a primary antibody to human type I or type III collagen for 60 min at room temperature. Both antibodies were used at a concentration of 1:200 [v/v] diluted in phosphate-buffered saline (PBS) with 0.1% [v/v] Tween 20 (PBST). Subsequently, the samples were incubated for 60 min at room temperature with a 1:500 dilution in PBST of AlexaFluor-labeled goat anti-mouse secondary antibody. Fluorescence images were obtained at an excitation of 473 nm and emission of 490–520 nm using the confocal microscope (Olympus).

### Statistical analysis

Values stated are mean ± SEM of the number of observations (n) indicated. Analysis of statistical significance was performed using a two-tailed Student's *t-*test. *p* < 0.05 was considered statistically significant.

## Results

### Construction of single and multilayered blood vessel models

**FIG. 2.**
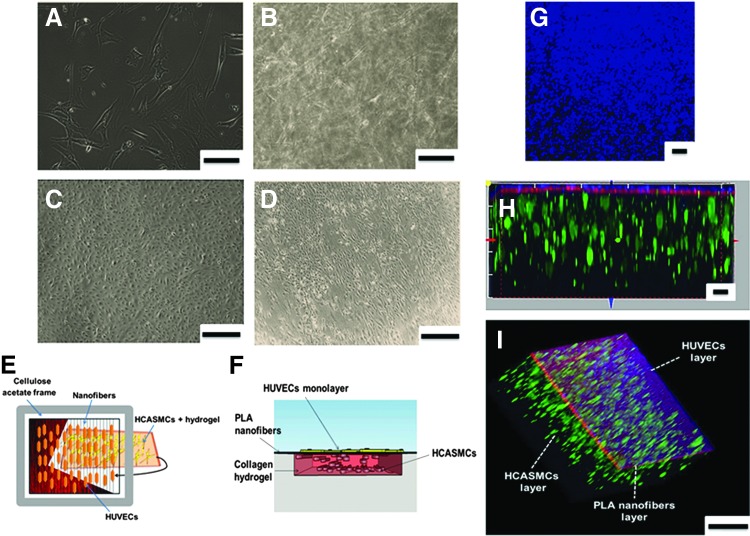
The morphology and assembly of the cells within the intimal and medial layer constructs. **(A)** Typical brightfield image of HCASMCs in monolayer culture. **(B)** Brightfield image of HCASMCs seeded and cultured in type I collagen hydrogel for 10 days. **(C)** Brightfield image of HUVECs in monolayer culture showing a typical cobblestone morphology of HUVECs. **(D)** Brightfield image of HUVECs in a full tissue-engineered blood vessel construct cultured for 4 days. The cells were grown on top of high-density, rhodamine-labeled aligned PLA nanofibers supported by a medial layer. **(E)** A diagrammatic representation of a *top* view of a multilayered TEBV construct and its assembly. **(F)** Side view sketch of the TEBV construct showing the locations of the cells within the construct. **(G)** An upper view image of fluorescently tracked HUVEC layer of the TEBV. **(H, I)** Side and luminal view images of the three distinct layers of TEBV construct. HUVECs (*blue*), PLA nanofibers (*red*), and HCASMCs (*green*). Scale bar **(A, B)** 150 μm; **(C, D)** 400 μm; **(G–I)** 30 μm. HCASMC, human coronary artery smooth muscle cell; HUVEC, human umbilical vein endothelial cell; TEBV, tissue engineering blood vessel. Color images available online at www.liebertpub.com/tec

TEML constructs were produced by seeding HCASMCs in type I collagen hydrogels. HCASMCs were initially cultured as a monolayer and acquired a spindle-shaped morphology (Fig. 2A). The cells were then harvested and seeded into type I collagen hydrogels. The cells rapidly regained their original spindle-shaped morphology within 24 h of seeding within the collagen hydrogels (Fig. 2B). The TEML was then cultured and cell morphology was monitored for up to 10 days.

A TEIL was constructed by culturing HUVECs atop an aligned PLA nanofiber mesh coated with fibronectin. HUVECs were initially cultured as a monolayer and acquired a cobblestone morphology (Fig. 2C). These cells were then harvested and seeded on top of the fibronectin-coated aligned nanofiber mesh supported with an underlying collagen hydrogel to create TEILs. The underlying collagen gel provided sufficient mechanical strength to allow easy handling of the constructs. The HUVECs were observed to be highly aligned within an hour postseeding (Fig. 2D) with a strong CD31 expression, indicating that the current culture condition maintained a normal endothelial cell phenotype (Supplementary Fig. S1; Supplementary Data are available online at www.liebertpub.com/tec).

To accurately replicate the physiological vessel wall layers, HUVECs were seeded atop aligned PLA nanofibers supported with the previously constructed TEML to create a 3D TEBV model (schematic shown in Fig. 2E, F). These cells were observed to show high alignment within an hour postseeding with HUVECs. From tracking the TEBV in coculture, it has been found that this technique has allowed us to reproducibly generate a bilayer vessel model, which maintained stably defined intimal and medial layers throughout the culture period without detachment (Fig. 2G–I).

### Detection of pro- and antiaggregatory properties of 3D models using [Ca^2+^]_cyt_ measurement

The pro- and antiaggregatory properties of TEML, TEIL, and TEBV models were simultaneously examined by measuring thrombin-evoked rises in [Ca^2+^]_cyt_ of platelets and examining their adhesion and aggregation on the luminal surface before thrombin stimulation. Constructs were individually placed atop the washed human platelet suspension for 15 min at 37°C with continuous magnetic stirring. This was followed by the removal of the constructs to allow imaging of the luminal surface of the construct. This initial phase allows us to examine the ability of platelets to adhere and aggregate upon the surface of the construct. To assess the cumulative effect of soluble agonists and inhibitors released from the 3D models, the platelet suspension was exogenously stimulated with 0.2 U/mL thrombin. Activation in the platelet suspension was assessed through real-time monitoring of Ca^2+^ signals.

#### Pro- and antiaggregatory properties detected by [Ca^2+^]_cyt_ in platelet suspensions

**FIG. 3.**
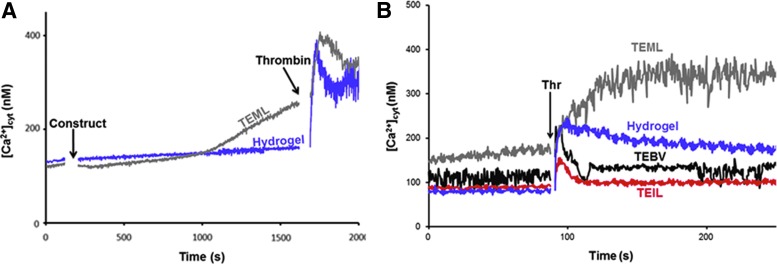
Real-time measurements of cytosolic calcium concentration [Ca^2+^]_cyt_ in platelet suspension exposed to acellular collagen hydrogel and tissue-engineered vessel constructs. Fura-2-loaded human platelets were exposed to the luminal surface of TEML or acellular hydrogel for 15 min at 37°C **(A)**, then the constructs were removed, and the [Ca^2+^]_cyt_ in the remaining suspension was recorded and stimulated with 0.2 U/mL thrombin **(B)**. Post-thrombin stimulation of all tissue-engineered constructs was recorded following a 15-min preincubation performed offscreen. Results are representative of four experiments. TEML, tissue-engineered medial layer; TEIL, tissue-engineered intimal layer; TEBV, tissue-engineered blood vessel; Thr, thrombin. Color images available online at www.liebertpub.com/tec

Thrombin-evoked changes in [Ca^2+^]_cyt_ of platelets preincubated with the different 3D constructs are shown in Figure 3A, B. Before thrombin stimulation, platelet exposure to the TEML was found to trigger a significant increase in cytosolic Ca^2+^ concentration compared with the cell-free collagen hydrogel (from 10 min in Fig. 3A and from 0 to 60 s in Fig. 3B). Subsequent stimulation with thrombin was found to elicit a greater increase in the [Ca^2+^]_cyt_ of platelets exposed to the TEML when compared with the collagen hydrogel alone (169.2% ± 33.1% of control; *n* = 8; *p* < 0.05). The higher [Ca^2+^]_cyt_ observed before thrombin stimulation is likely due to activation of the platelets through binding to adhesive ligands on the surface of the TEML and releasing autocrine signaling molecules from their dense granules, which can trigger platelet activation in the washed platelet suspension.

In these experiments, the washed platelet suspension was prepared using aspirin to remove the potential for autocrine activation of platelets by thromboxane A2 production. This can significantly potentiate any agonist-evoked rise in [Ca^2+^]_cyt_ and thus interfere with the study of primary activation mechanisms of platelets by soluble or adhesive agonists.^14^ An alternative method to prepare washed platelet suspensions is through treatment with prostacyclin to help keep platelets in a quiescent state. Experiments comparing these methods of platelet preparation showed no significant differences (Supplementary Fig. S2), suggesting that the method of platelet preparation will not alter the signals observed.

In contrast to our results with the TEML, exposure of platelets to the full 3D TEBV construct elicited no significant rise in [Ca^2+^]_cyt_ in isolation. However, this exposure was found to reduce platelet responsiveness to thrombin simulation, as could be seen by significant reduction in the subsequent thrombin-evoked rise in [Ca^2+^]_cyt_ (24.0% ± 11.3% of control; *n* = *8; p* < 0.05). Similarly, the exposure of washed platelets to the TEIL causes a significant reduction of thrombin-evoked [Ca^2+^]_cyt_ when compared with samples exposed to the acellular collagen hydrogel. (24.9% ± 13.7% of control; *n* = *8; p* < 0.05). At 120 s, the relative degree of [Ca^2+^]_cyt_ increase observed in response to thrombin stimulation was as follows: TEIL<TEBV<collagen<TEML.

#### Pro- or antiaggregatory properties detected by platelet aggregation

**FIG. 4.**
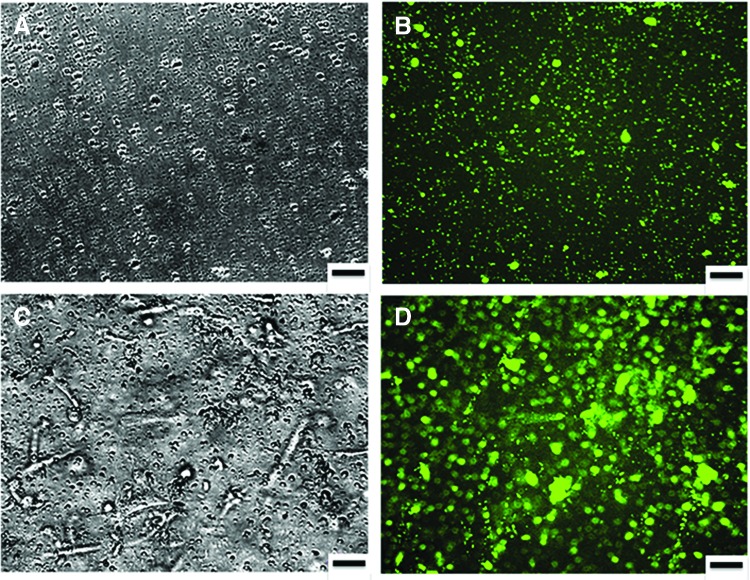
Optical images of acellular collagen hydrogel and TEML constructs after exposure to platelets. DiOC_6_-labeled human platelet suspension was incubated with type I collagen hydrogel **(A, B)** or a TEML **(C, D)** for 15 min at 37°C with gentle agitation of samples. Platelet aggregation was recorded, after the samples were washed with HBS, under a brightfield microscope **(A, C)** and fluorescence microscope **(B, D)**. Results presented are representative of four experiments. Scale bar = 50 μm. HBS, HEPES-buffered saline. Color images available online at www.liebertpub.com/tec

Washed, DiOC_6_-labeled platelet suspensions were allowed to interact with an acellular type I collagen hydrogel or the TEML. Fluorescence imaging of the DiOC_6_-labeled platelet-exposed surface of the constructs revealed that the acellular collagen hydrogels only possessed sporadic platelet adhesion and there were no obvious signs of any platelet aggregates forming upon the surface (Fig. 4A, B). In contrast, the TEML construct was found to support greater platelet adhesion as well as the formation of some platelet aggregates (Fig. 4C, D).

### Collagen fibril morphology

**FIG. 5.**
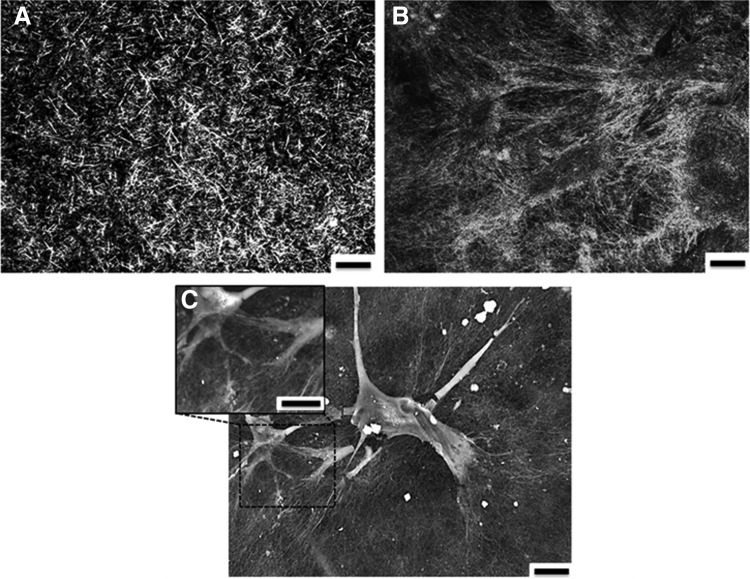
Collagen fibrillar morphology in acelular collagen hydrogel and TEML. Confocal reflection images of acellular collagen hydrogel **(A)** and TEML construct **(B)** cultured for 10 days. Scale bar = 10 μm. **(C)** SEM image of the same TEML sample. Scale bar 50 μm. Results are representative of five experiments. SEM, scanning electron microscopy.

Reflectance confocal microscopy was utilized to examine the presence of neo-collagen secreted by HCASMCs within the TEML and the difference of the collagen fibril morphology between the neo-collagen and scaffolding collagen. As shown in Figure 5A and B, distinct architectural differences could be observed in the TEML compared with those not seeded with HCASMCs. More specifically, the type I collagen fibrils observed in the acellular hydrogel exhibited a larger diameter and a greater degree of shorter fibrils compared with hydrogels in which HCASMCs were cultured. Additionally, fibrils of the TEML construct appeared more densely packed compared with those of the reconstructed hydrogels without HCASMCs.

Further examination of the TEML using SEM demonstrated that the HCASMCs have created a pericellular matrix consisting of a dense mesh of fibrils, which extended from the surface of the cell in an organized manner (Fig. 5C). These highly arranged collagen fibrils emerging from the cells exhibited different morphology in comparison with fibrils far from the cell body, which were from reconstructed type I collagen hydrogel.

**FIG. 6.**
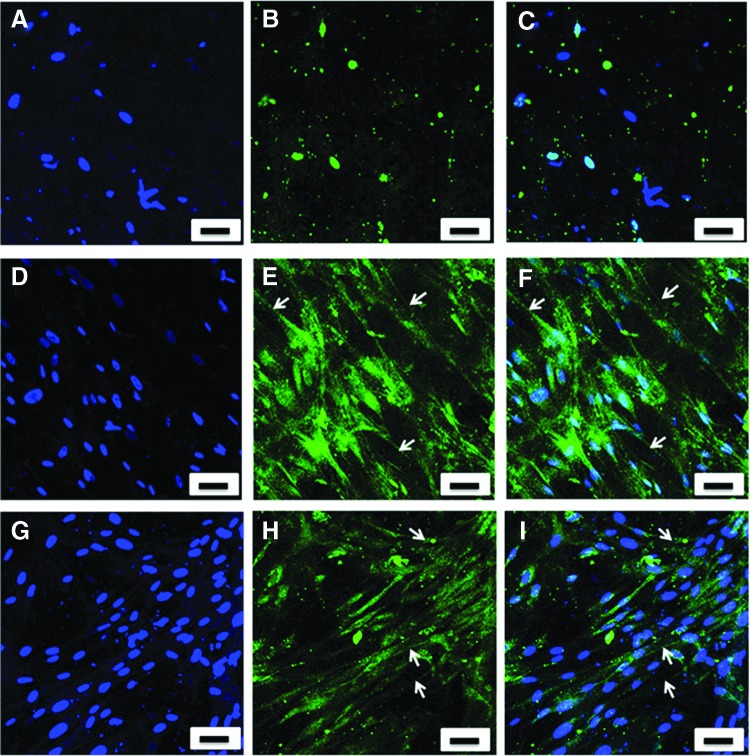
Immunohistochemical staining of collagen types I and III within the TEML samples. Primary antibody-free control **(A–C)**; collagen type I in TEML sample cultured for 10 days **(D–F)**; collagen type III in TEML sample cultured for 10 days **(G–I)**. **(A, D, G)** show DAPI-staining alone; **(B, E, H)** show images of the antibody staining alone; **(C, F, I)** shows the overlay of both images. The results presented are representative of four experiments. Scale bar 50 μm. Color images available online at www.liebertpub.com/tec

The TEML constructs and collagen hydrogel were examined by confocal microscopy after being labeled with a fluorescently tagged antibody to type I or type III human collagen. There was no binding of the antibody to the acellular collagen hydrogel, while there was strong fluorescence found to occur around the HCASMCs within the hydrogel for both types of collagen (Fig. 6).

## Discussion

### A novel testing platform for quantitatively assessing the ability of tissue-engineered blood vessel constructs to modulate platelet activation status

In this study, we have demonstrated a novel testing platform to assess the physiological functions of the tissue-engineered blood vessel constructs through analyzing the effect on platelet activation status (Fig. 1). Through exposing the constructs to washed human platelet suspension colabeled with both Fura-2 and DiOC_6_, we are able to simultaneously assess the ability of the platelet to adhere and aggregate upon the surface of the vessel constructs, real-time monitoring of the activation status of the platelet suspension, or modulation of platelet responsiveness to soluble agonists. This ability to assess the cumulative effect of platelet agonists and inhibitors in a single simple assay provides the basis for a standardized and quantifiable method to directly compare effects of a range of different tissue-engineered vessel constructs on platelet activation. As such, this system may also be suitable for use as a standardized *in vitro* testing system for comparing the pro- and antiaggregatory properties of tissue-engineered blood vessel constructs cultured in different ways. This may facilitate the development of culturing methods, which allow the development of optimal antiaggregatory properties of the intimal layer and proaggregatory properties of the medial layer.

### The testing platform can quantitatively assess the proaggregatory capacity of distinct collagen-containing scaffolds

A notable feature of our work was the finding that the acellular type I collagen hydrogel was unable to support significant platelet activation and aggregation when exposed to a washed human platelet suspension (Fig. 4A, B). Previous studies have demonstrated that collagen structure is vital for receptor–collagen interaction and thus its ability to elicit effective platelet activation.^21,22^ Type I collagen monomers can spontaneously self-assemble into fibrils,^23^ whose structure is heavily dependent upon the gelation conditions used and the intactness of the N- and C-telopeptides, as well as the presence of other types of collagen and proteoglycans.^24^ Although the presence of collagen fibrils could clearly be seen in our reflectance microscopy studies of the acellular hydrogels, this form of collagen was clearly unable to trigger a significant change in platelet activity (Fig. 5A). This may be related to a failure of the self-assembly process to fully recreate the tertiary and quaternary structures of collagen found in native collagen forms, or instead may be related to differences in composition from the native forms.

In contrast to the acellular type I collagen hydrogels alone, the presence of HCASMCs within the hydrogel was shown to lead to the production of neo-collagen able to support significant platelet activation both upon the surface of the construct and sensitizing the remaining platelets in suspension to exogenous agonists (Fig. 5B, C). The immunochemical staining of our TEML was consistent with the well-established observation that cultured smooth muscle cells are able to produce and secrete both type I and type III collagen (Fig. 6).^25,26^ Similarly, this mixture of collagen forms is also found within the native blood vessel as well as within Horm collagen (a native form of collagen widely used to trigger strong platelet aggregation in *ex vivo* platelet function testing).^27^ These results confirm that although type I collagen is the main isomer on the surface of the medial layer, which can mediate platelet activation, there are clearly significant higher-order structural differences between the neo-collagen produced by the HCASMCs and that in the collagen hydrogel, which underlie their differing efficacy in mediating platelet activation. These differences may be due to differences in the collagen fibril structure, composition (neo-collagen is a mixture of type I and type III), the presence of nonhelical regions, and the degree of cross-linking in the fibers of these two collagen sources—all of which are known to be essential in collagen's ability to trigger platelet aggregation.^28–30^ Our nonaggregatory type I collagen scaffold and proaggregatory TEML provide useful systems in which to further study the role of collagen type I structure in mediating platelet activation.

### Tissue-engineered blood vessel models as a tool for *ex vivo* human platelet function testing

In this work, we have utilized our testing platform to test the effect of our intimal, medial, and blood vessel constructs on platelet adhesion and aggregation upon the vessel surface. These studies have demonstrated the feasibility of creating intimal, medial, and full blood vessel constructs, which replicate their *in vivo* ability to alter platelet function. Both the intimal layer and full constructs prevented platelet activation in line with the known antiplatelet properties of the endothelial lining (Fig. 3). In contrast, the HCASMC-containing medial layer was found to support effective platelet activation and aggregation–in line with these constructs' ability to mimic the normal primary hemostasis reactions found in the damaged blood vessels.^31^ These results therefore suggest the possibility that such tissue-engineered constructs could be used to improve the physiological validity of *ex vivo* studies of platelet activation pathways and assessment of platelet function such as Ca^2+^ signaling,^32^ a commonly used method. In addition, it should be possible to adapt this system for use with other functional assays (e.g., granule secretion assays or assessment of procoagulant activity).

## Conclusion

In this article, we introduce a novel testing platform to examine platelet activation upon exposure to either full or partial tissue-engineered blood vessel constructs. Using this system, we have been able to demonstrate that while reconstituted type I collagen hydrogels have limited capacity to support platelet activation, the neo-collagen generated by HCASMCs within the constructs has a strong proaggregatory capacity. By incorporating an intimal layer on top of these structures, it is able to create a luminal surface, which prevents the proaggregatory nature of the medial layer, and mimics the antiplatelet properties of the tunica intima found *in vivo*. These data demonstrate that the methodology presented here is able to quantitatively compare the capacity of different constructs to trigger or prevent platelet activation. As such, this technique may provide a useful tool for standardizing the assessment of functional properties of tissue-engineered blood vessel constructs developed using different culturing techniques.

## Supplementary Material

Supplemental data

Supplemental data
