# Supplementary material for: A Real-Time Monitoring System to Assess the Platelet Aggregatory Capacity of Components of a Tissue-Engineered Blood Vessel Wall
Source: Tissue Eng Part C Methods. 2016 Jun 24;22(7):691–9. doi: 10.1089/ten.tec.2015.0582 (PMC4943470; doi:10.1089/ten.tec.2015.0582)
Supplement: Supplemental data [file Supp_Fig1.pdf]

Supplementary Data

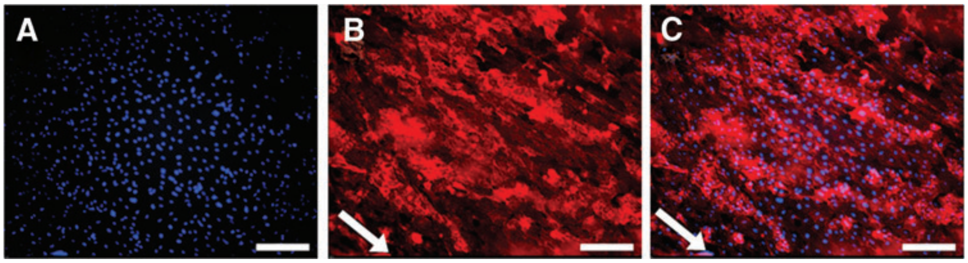

SUPPLEMENTARY FIG. S1. Fluorescent image of CD31 expression of HUVECs grown and culture in TEIL with PLA nanofibers as the basement membrane. **(A)** DAPI (nucleus, *blue*), **(B)** CD31 (HUVEC marker, *red*), **(C)** overlay image of **A** and **B**. The *arrows* indicate the alignment direction in nanofiber. Scale bar = 200 μm. HUVEC, human umbilical vein endothelial cell; TEIL, tissue-engineered intimal layer.
