# Supplementary material for: A Real-Time Monitoring System to Assess the Platelet Aggregatory Capacity of Components of a Tissue-Engineered Blood Vessel Wall
Source: Tissue Eng Part C Methods. 2016 Jun 24;22(7):691–9. doi: 10.1089/ten.tec.2015.0582 (PMC4943470; doi:10.1089/ten.tec.2015.0582)
Supplement: Supplemental data [file Supp_Fig2.pdf]

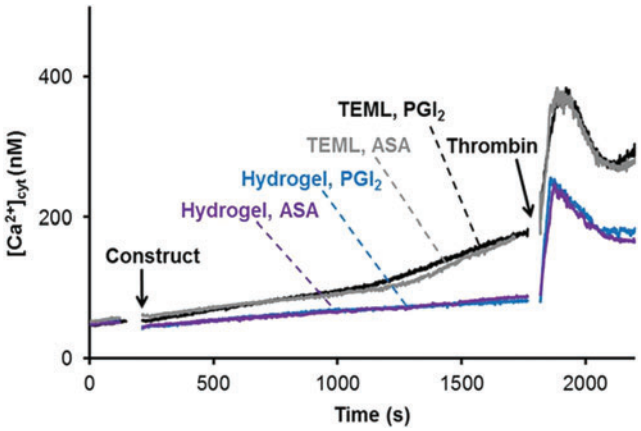

SUPPLEMENTARY FIG. S2. Real-time measurements of cytosolic calcium concentration $[Ca^{2+}]_{cyt}$ in the wash platelet suspension prepared by aspirin (ASA) or prostacyclin (PGI_2) exposing to acellular collagen hydrogel and TEML vessel constructs for 15 min at 37°C. The constructs were removed and the $[Ca^{2+}]_{cyt}$ in the remaining suspension was recorded and stimulated with 0.2 U/mL thrombin. Results are representative of four experiments. TEML, tissue-engineered medial layer.
